# Physeal Bystander Effects in Rhabdomyosarcoma Radiotherapy: Experiments in a New Xenograft Model

**DOI:** 10.1155/2011/815190

**Published:** 2011-04-17

**Authors:** Jason A. Horton, Judith A. Strauss, Matthew J. Allen, Timothy A. Damron

**Affiliations:** ^1^Department of Orthopaedic Surgery, Musculoskeletal Sciences Research Center, Institute for Human Performance, SUNY Upstate Medical University, 505 Irving Avenue, Syracuse, NY 13210, USA; ^2^Imaging and Molecular Therapeutics Section, Radiation Oncology Branch, National Cancer Institute, National Institutes of Health, 10 Center Drive, Room B3B100, MSC 1002, Bethesda, MD 20892-1002, USA; ^3^Department of Veterinary Clinical Sciences, Veterinary Medical Center, The Ohio State University, 601 Vernon Tharp Street, Columbus, OH 43210, USA

## Abstract

Radiotherapy used in the treatment of pediatric musculoskeletal sarcomas may result in crippling defects of skeletal growth. Several radioprotective strategies have shown potential for preserving function of the irradiated epiphysis but have not been evaluated in a tumor-bearing animal model. We developed two bioluminescent human rhabdomyosarcoma cell lines that were used to establish xenograft tumors in skeletally immature mice. Bioluminescence imaging and radiography allowed serial evaluation of tumor growth and tibial elongation following localized radiotherapy. High-dose (10 Gy) radiotherapy significantly reduced tumor growth velocity and prolonged the median survival of tumor-bearing mice but also resulted in a significant 3.3% shortening of the irradiated limb. Exposure to a lower, 2 Gy dose resulted in 4.1% decrease in limb length but did not extend survival. This new model provides a clinically relevant means to test the efficacy and safety of novel radioprotectant and radiorecovery strategies for use in this context.

## 1. Introduction

Rhabdomyosarcoma is the most common pediatric soft-tissue sarcoma [1]. It is a malignant tumor thought to derive from the skeletal muscle lineage that primarily affects young children and adolescents, and very rarely adults [2]. A multimodal approach to rhabdomyosarcoma treatment is favored, where surgical excision and chemotherapy are used in conjunction with external-beam radiotherapy to achieve tumor eradication [3, 4]. Skeletal complications including limb length asymmetry, crippling angular deformity, and pathologic fracture may result when the irradiated field is inclusive of the epiphyseal growth plate [5, 6]. Laboratory studies have demonstrated the efficacy of radioprotectant compounds such as amifostine in reducing the injurious effects of ionizing radiation to the skeletal growth plate, but due to the lack of a clinically relevant, tumor-bearing animal model have not yet been tested for use in this context [7–9]. 

Commonly, preclinical testing of antitumor and supportive therapies involve inoculation of human tumor cell lines into immunocompromised murine hosts [10]. The utility of this approach is further enhanced by the application of *in vivo* molecular imaging technologies, allowing serial assessment of response to experimental treatments. In this report, we have developed a model system for the study of pediatric rhabdomyosarcoma in general and the evaluation of adverse bystander effects of radiotherapy on the skeletal growth plate in particular. The specific aims of the current study were to (1) establish variants of two widely-studied rhabdomyosarcoma cell lines, which stably express a luciferase tracer, (2) use these cells to develop an orthotopic xenograft model of rhabdomyosarcoma in NCr nude mice that allows the evaluation of tumor progression by bioluminescence imaging, and (3) to document the adverse skeletal sequelae of irradiating an orthotopic tumor in growing mice. It was hypothesized that intramuscular rather than subcutaneous implantation of human rhabdomyosarcoma tumor cells would result in more robust tumor engraftment and growth. We then hypothesized that the use of external beam radiotherapy would reduce orthotopic tumor growth and prolong survival. It was anticipated that the irradiation of the tumor bed and the adjacent epiphyseal growth plate would result in significant limb length asymmetry.

## 2. Methods

### 2.1. Rhabdomyosarcoma Tumor Cell Lines

Two well-established rhabdomyosarcoma cell lines were used in this series of experiments. The embryonal variant is represented in our studies by the cell line RD (ATCC# CCL-136, Manassas, Va, USA). The cell line RC13, also referred to variably in the literature as SJCRH30, RC-13, RMS 13, SJRH30, or RH30 (ATCC #CRL-2061), is representative of the alveolar variant. Both cell lines were cultured in DMEM, supplemented with 10% v/v heat-inactivated fetal calf serum, 4.5 mg/mL glucose, 4 mM L-glutamine, 50 IU/mL penicillin, 50 *μ*g/mL streptomycin, 10 mM HEPES, and 1 mM sodium pyruvate. All cell culture products were obtained (from CellGro, Manassas, Va, USA). Both cell lines express MyoD1 and myogenin at the transcript and protein level, which are markers of early myogenic differentiation but that are absent in mature skeletal muscle. The RC13 cells also express a fusion transcript corresponding to the *t*(2:13) translocation joining the Pax3 and FOXO1 (formerly Fkhr) genes that are a diagnostic characteristic of alveolar rhabdomyosarcoma [11]. For validation purposes, immunochemistry was used to verify the expression of these markers at the protein level in both cell lines, while RT-PCR was used to demonstrate and verify the expression of the Pax3:FKHR fusion transcript in the RC13 cells (data not shown).

### 2.2. Transfection and Selection of Bioluminescent Rhabdomyosarcoma Lines

Lipofectamine LTX/Plus (Invitrogen, Carlsbad, Calif, USA) was used to transfect 1 × 10^5^ RD or RC-13 cells with the 10 *μ*g of plasmid vector pEGFP-Luc (Clontech, Mountainview, Calif, USA). This vector allows the expression of enhanced green fluorescent protein:firefly luciferase (EGFP-Luc) fusion protein under the control of the human cytomegalovirus (CMV) promoter element and allows antibiotic selection with G418 by virtue of a neomycin-resistance cassette regulated by an SV40 promoter. Following transfection, cells were plated in 10 cm dishes and incubated in growth media supplemented with 500 *μ*g/mL G418 for 10 days to select neomycin-resistant clones. Individual colonies of each transfected cell line were isolated, examined for EGFP fluorescence, harvested with trypsin, and expanded for 10 passages in the presence of 200 *μ*g/mL G418 to establish stable expression of the EGFP-Luc fusion protein, yielding the clones RD-Luc and RC13-Luc. Prior to inoculation into murine hosts, we verified the expression of the EGFP-Luc fusion transcript by RT-PCR, EGFP expression by epifluorescence microscopy, and luciferase activity by an *in vitro* luciferase assay system (Promega, Madison, Wis, USA). For xenograft experiments, tumor cells were grown to approximately 50–75% confluence prior to harvesting with 0.05% trypsin/0.53 mM EDTA, centrifuged, and resuspended in Hank's buffered salt solution (HBSS) at 1 × 10^8^ cells/mL. This suspension was further diluted by the addition of an equal volume of ice-cold HBSS or ice-cold growth-factor reduced matrigel (BD Biosciences, San Jose, Calif, USA), and incubated on ice until inoculation into animal hosts.

### 2.3. Murine Xenograft Experiments

All experimental procedures involving the use of animals were performed in accordance with a protocol approved by the local IACUC. A total of *n* = 66 weanling (21 days old) homozygous male NCr nude mice (CrTac:NCr-Foxn1^nu^, Taconic Farms Inc., Germantown, NY, USA) were obtained for all studies and randomized to experimental groups as detailed in Table 1. During routine husbandry, all mice were maintained in an aseptic isolation colony in groups of *n* = 3 mice per cage, with 12-hour light/dark cycles and *ad libitum* access to sterilized chow and water. Anesthesia during tumor inoculation was by an intraperitoneal injection of a combination of tiletamine and zolazepam (Telazol, 15 mg/kg; Fort Dodge Animal Health, Fort Dodge, Iowa, USA). For ectopic inoculations, anesthetized animals were positioned prone, and a 22-gauge needle was inserted subcutaneously over the right flank and swept to create a pocket prior to the delivery of 20 *μ*L of the cell suspension. For tumor-naïve animals, an equivalent cell-free suspension was prepared and held on ice prior to injection. For orthotopic intramuscular inoculations, the mouse was positioned supine with the right limb extended; the anterior-distal quadriceps was palpated, and 20 *μ*L of the cell suspension was delivered through a 22 g needle inserted into the thigh musculature. Following inoculation, animals were returned to their cages and allowed to recover from the anesthetic. Two mice from the sham-irradiated control group succumbed during anesthesia; no other complications or infections were observed. Subsequently, the mice were monitored daily for general health and well-being. On a weekly basis, animals were weighed and examined by palpation of the inoculation site and ipsilateral lymph nodes. General malaise, weight loss greater than 10%, or tumor mass approximating 20% of body weight was criteria for euthanization in accordance with institutional protocol.

### 2.4. *In Vivo* Bioluminescence Imaging

**Figure 1:**
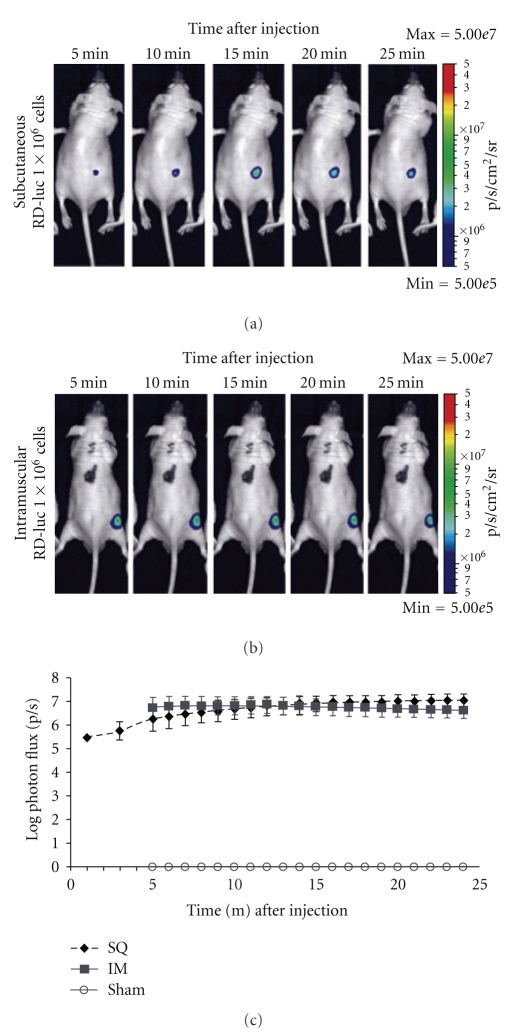
Luciferin uptake and signal stabilization. Bioluminescence imaging was used to verify tumor placement three hours after (a) subcutaneous or (b) intramuscular inoculation of RD-luc cells. Imaging was initiated one-minute following a single, 150 mg/kg I.P. bolus of d-luciferin mice, and photon flux data was collected in successive one minute intervals. (c) Average log-photon flux ±1 SD of *n* = 4 mice demonstrating a detectable signal within 1 minute following luciferin injection and was stable for a period ranging from 10 to 24 minutes after-injection. No signal was detected in animals injected with cell-free Matrigel.

Imaging studies were performed immediately after inoculation and on a weekly basis thereafter using the IVIS-50 instrument (Caliper Life Sciences, Hopkinton, Mass, USA). The mice were anesthetized by isoflurane inhalation, and injected intraperitoneally with 150 mg/kg d-Luciferin potassium salt dissolved in normal saline (Caliper). An initial calibration experiment demonstrated that the luciferase signal reached a plateau, approximately 8–10 minutes following d-luciferin injection, and remained stable for at least 14 minutes thereafter (Figure 1). All subsequent imaging studies were initiated 10 minutes following the injection of d-luciferin, with photon flux (photons/second) data collected for a period of 5 minutes. Imaging data analysis was performed using Living Image v3.2 software (Caliper Life Sciences).

### 2.5. External Beam Radiotherapy

Prior to irradiation, mice were anesthetized with an intraperitoneal injection of Telazol (15 mg/kg) and positioned on a Plexiglas platform with the right leg, inclusive of the tumor bed, extended through a 2 cm × 4 cm collimated radiation field. Lead shielding was used to protect the remainder of the body from direct or scattered radiation. The animals were then positioned beneath the radiation unit (Phillips MGC-30, Farmington, Conn, USA) at a source-to-skin distance of 30 cm, delivering 300 kVp/10 mA X-rays, at an effective dose rate of 2.56 Gy/min. Groups of *n* = 6 mice received single exposures of either 2 Gy or 10 Gy, with the remaining *n* = 4 mice in the 0 Gy control group anesthetized and positioned in the same manner as their irradiated cohort.

### 2.6. *Ex Vivo* Analyses of Normal and Tumor Tissues

At the conclusion of the experimental period, or when physical examination warranted humane termination, animals were euthanized by CO_2_ inhalation, and death was verified by the absence of a cardiac pulse. The visceral organs and lymph nodes were examined grossly for systemic dissemination, and affected tissues were preserved in 10% neutral buffered formalin. Tumor tissues were excised, and segments were fixed in 10% neutral buffered formalin for histology and immunohistochemistry. Tissues adjacent to the inoculation site were processed similarly for mice where no tumor growth was evident. Following excision of the tumor tissue, the hind limbs were disarticulated at the hip, radiographed in the sagittal plane, and fixed in 10% neutral buffered formalin. After 24 hours of fixation at 4^*◦*^C, the tissues were rinsed in phosphate-buffered saline and decalcified with daily changes of 10% EDTA over a period of two weeks. Subsequently, all tissues were dehydrated through ascending ethanol series, cleared in xylene, and embedded in paraffin. Tissue sections were then cut at 5 *μ*m thickness and stained with hematoxylin and eosin for histopathologic examination or probed with antibodies directed against human myogenin (#sc-319434, Santa Cruz Biotechnology, Santa Cruz, Calif, USA) or myoD1(#ab16148 AbCam, Cambridge Mass, USA) antigens following retrieval in boiling citrate buffer. Positive immunoreactivity was subsequently detected with a biotinylated secondary antibody (Vectastain ABC Elite-Universal, Vector Labs Burlingame, Calif, USA) and visualized by ImmPRESS DAB histochemistry, Vector Labs, Burlingame, Calif, USA).

Baseline and terminal lengths of the tibia and femur were measured for each mouse in the radiotherapy experiment, from radiographs taken prior to inoculation and immediately following euthanasia. Femoral length was measured perpendicular to a line extending from the inter-condylar notch distally and through the apex of the greater trochanter proximally. Tibial length was measured perpendicular to a line extending from the apex of the tibiotalar cup distally to the center of the tibial plateau proximally. Limb length was then taken as the sum of the tibia and femur measurements for each animal, and growth was taken as the difference in length between the initial and terminal lengths.

### 2.7. Statistical Analysis

Raw photon flux (p/s) data were log-transformed prior to statistical analysis. Differences in limb length between irradiated and nonirradiated limbs were assessed by the Student's paired *t*-test, and ANOVA was used to examine differences between the radiation-dose groups, accepting differences as statistically significant when *P* ≤ .05. Median survival time after-inoculation was evaluated by Mantel-Cox Log-Rank test. All statistical analyses were performed using StatView software v5.01 (SAS Institute, Cary, NC, USA).

## 3. Results

### 3.1. Comparison of Tumor Engraftment and Growth in Ectopic versus Orthotopic Locations

Engraftment of RD cells injected as a suspension in an aqueous vehicle at titers of 1 × 10^4^, 1 × 10^5^, or 1 × 10^6^ cells/mouse did not result in any detectable tumor engraftment by palpation or by histological examination of the inoculated musculature or ipsilateral lymphatic chain tissues in a 12-week pilot study. The failure to achieve engraftment using aqueous media prompted our decision to suspend the tumor cells in Matrigel. To further enhance our ability to detect occult tumors and metastases, we established stably transfected variants of two human rhabdomyosarcoma cell lines expressing firefly luciferase, RD-luc, and RC13-luc, to permit the use of bioluminescence imaging. This approach allowed the visualization of the tumor cell mass as early as three hours after inoculation (Figure 1). These adaptations greatly improved the likelihood of detectable engraftment to greater than 97% (Table 1).

### 3.2. Bioluminescence Imaging Facilitates Tumor Localization and Measurement

**Figure 3:**
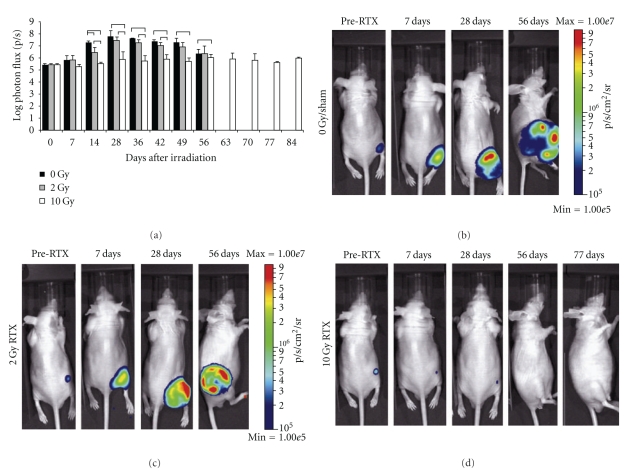
Serial bioluminescence imaging demonstrating successful tumor engraftment resulting from subcutaneous and intramuscular inoculation of RD-luc and RC13-luc tumor cells. (a) The growth of RD-luc tumors did not demonstrate substantial tumor growth when implanted either (b) subcutaneously or (c) intramuscularly. (d) Modest growth of RC13-luc cells were implanted (e) subcutaneously, whereas aggressive tumor growth resulted when this cell line was inoculated intramuscularly. Bar graphs show log-photon flux ±1 SD; brackets indicate *P* ≤ .05 by Student's *t*-test.

We then followed the growth rate of xenografts derived from inoculation of RD-luc or RC13-luc cells suspended in Matrigel when inoculated in an ectopic (subcutaneous) or orthotopic location (intramuscular). Regardless of the location, RD-luc cells persisted at the inoculation site but failed to produce significant (*P* > .2667) tumor growth by 9 weeks after inoculation (Figures 3(a)–3(c)). Luminescence of the orthotopically placed RD-Luc cells was significantly greater (*P* = .0172) than ectopic subcutaneous tumors at 26 days after inoculation, but differences at all other time points were not statistically significant (*P* > .2470). 

By contrast, orthotopic inoculation of the RC13-Luc cell line formed a rapidly growing tumor, outwardly evident by 14 days after inoculation and necessitating humane euthanasia as early as 35 days after inoculation (Figures 3(d)–3(f)). RC13-Luc inoculation into the thigh musculature resulted in significantly larger tumors at each weekly time-point following inoculation (*P* > .0055) when compared to RC13-Luc cells inoculated subcutaneously. 

At the end of study, necropsies were performed and tumor tissues were preserved for histological examination. Ectopic inoculation of either cell line produced a firm, poorly vascularized mass that was loosely associated with subcutaneous fascia. Orthotopic inoculation of RD-luc cells produced small, firm tumors that were confined to the inoculated muscle compartment. In contrast, orthotopic inoculation of RC13-luc cells produced tumors that were infiltrating the underlying musculature and displayed prominent superficial vascularization, with regions of cystic necrosis centrally. Both cell lines demonstrated histological features, respectively, corresponding to the implanted embryonal or alveolar variants of rhabdomyosarcoma, including nests of small round blue cells with fibrovascular septae and positive MyoD1 and Myogenin immunochemistry. Injection of cell-free Matrigel into either location did not produce any signs of neoplasia, infection, inflammation, or lymph node enlargement and was not evident on histological examination of local tissues retrieved at the end of study.

### 3.3. Radiotherapy Inhibits Tumor Growth and Prolongs Survival but Results in Limb Length Asymmetry

**Figure 2:**
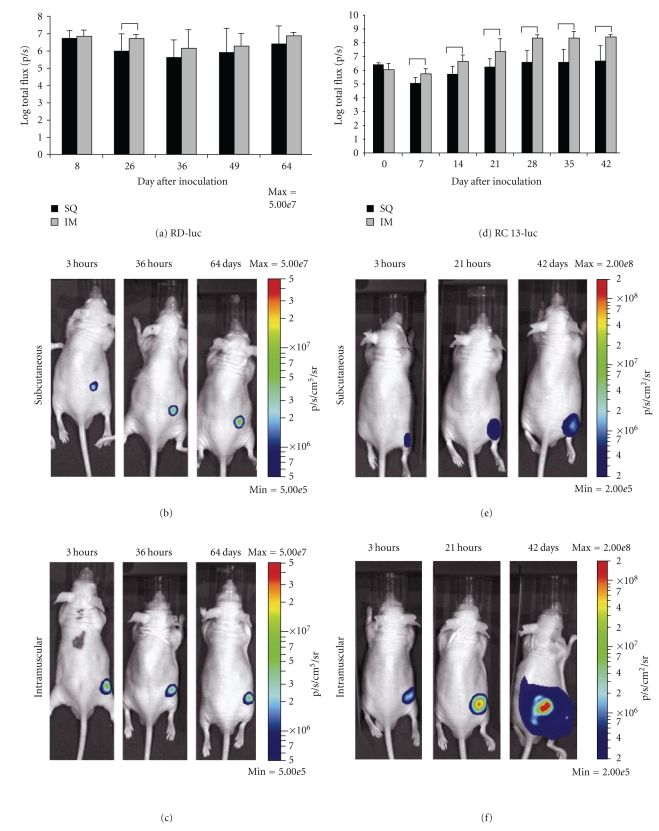
Serial bioluminescence imaging RC13-luc xenografts following radiotherapy with 0 Gy, 2 Gy, or 10 Gy X-rays. All animals in the 0 Gy and 2 Gy groups were euthanized within 63 days of imaging. (a) Data presented as the mean log-transformed photon-flux (photons/second) ±1 SD; brackets indicate *P* ≤ .05 by ANOVA with Bonferroni-Dunn post hoc test. Representative images demonstrating tumor localization and growth in (b) nonirradiated, (c) 2 Gyirradiated, and (d) 10 Gy irradiated mice.

Localized ionizing radiation therapy (0, 2, or 10 Gy X-rays at 300 kVp) was administered to the tumor-bearing limb over a field encompassing the distal femoral and proximal tibial growth plates. The remainder of the body including the non-tumor-bearing limb was protected from direct and scattered radiation by beam collimation and lead shielding. For our experiments, radiotherapy was initiated one week after inoculation, immediately following the demonstration of successful tumor engraftment by bioluminescence imaging. Prior to irradiation, no significant differences were observed between dose groups (Figure 2(a)). Nonirradiated tumors displayed aggressive growth necessitating humane euthanasia of all mice in this group within 64 days of inoculation. Localized radiotherapy of orthotopic RC13-Luc xenografts resulted in a dose- and time-dependent suppression of tumor growth (Figures 2(c) and 2(d)). Bioluminescence of tumors exposed to 2 Gy X-rays was significantly reduced in comparison to the sham-irradiated group through 42 days after inoculation, In contrast to the sham-irradiated group, 10 Gy irradiation significantly suppressed tumor growth through 49 days after inoculation. Statistically significant differences between the 2 Gy and 10 Gy groups were only apparent one week following radiotherapy. 

**Figure 4:**
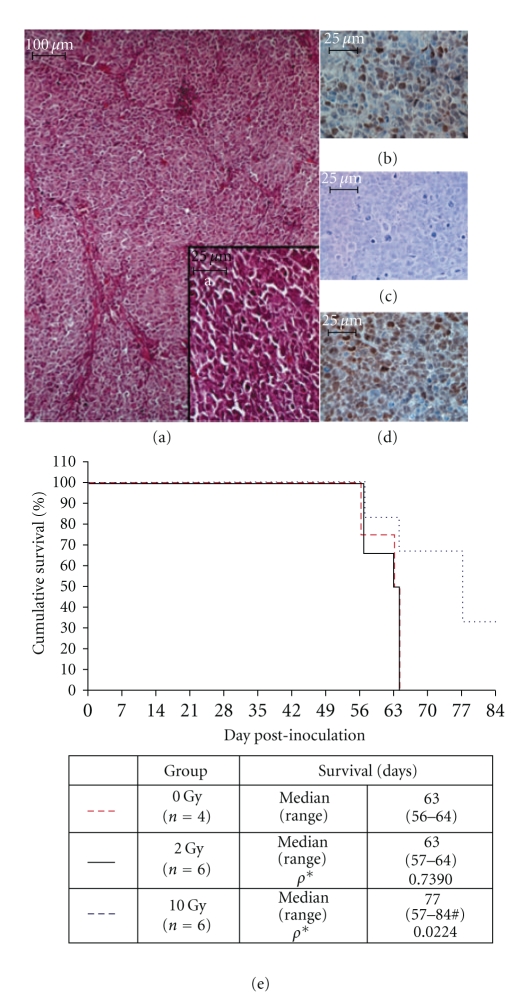
Representative histology and immunohistochemistry of RC13-luc derived metastatic lesion. (a) H&E, 10x, and (a) 40x; (b) MyoD1; (c) IHC neg. control; (d) Myogenin. (e) Cumulative survival. Kaplan-Meier plot of survival in tumor-bearing mice following localized irradiation with 2 Gy (solid black) or 10 Gy X-rays (dotted blue), or 0 Gy, sham-irradiated mice.

Aggressive tumor growth necessitated the termination of all mice in the sham-irradiated and 2 Gy-irradiated groups between 56 and 64 days after inoculation (Figure 4). Necropsy showed gross evidence of metastatic dissemination in three mice from the sham-irradiated group: one associated with the right kidney, one along the ureter, and one attached to the chest wall superior to the xiphoid. These distant masses appeared histologically consistent with the primary tumor and also displayed positive MyoD1 and myogenin immunoreactivity. (Figures 4(a)–4(d)) Four mice from the 10 Gy-irradiated group were euthanized due to tumor burden from days 56–77 after inoculation; however two mice from the 10 Gy-irradiated group presented only a locally contained lesion at 84 days without a sign of systemic dissemination. Median survival of sham-irradiated (range 56–64) and 2 Gy- (range 57–64) irradiated mice was 63 days after inoculation and was not significantly different (*P* = .7390). Median survival of 10 Gy irradiated mice (77 days, range 57–84 days) was significantly longer than that of non-radiated mice (*P* = .0224) (Figure 4(e)). 

Irradiation of the tumor bed and the underlying knee region inclusive of the distal femoral and proximal tibial growth plates, resulted in a reduction of the length of the irradiated limbs relative to the contralateral, nonirradiated limbs as measured from contact radiographs (Table 2). The average total length of the tumor-bearing limb of mice receiving radiotherapy was significantly reduced with respect to the contralateral control by 1.2 ± 0.2 mm (*P* = .0007) for the 2 Gy-treated mice and 0.9 ± 0.03 mm (*P* = .0067) in 10 Gy-irradiated mice. In contrast, the 0.3 ± 0.4 mm average difference between the control and tumor-bearing limbs in the sham-irradiated group was not significant (*P* = .3769). The total length of the nonirradiated limbs of 10 Gy-treated mice (30.4 ± 0.6 mm) was greater than that of the 2 Gy (29.1 ± 0.7 mm, *P* = .0049) and nonirradiated controls (28.6 ± 0.8 mm, *P* = .0015). Similarly, the tumor-bearing irradiated limb of the 10 Gy group (29.4 ± 0.8 mm) was significantly longer than the tumor-bearing limb in either the nonirradiated control group (28.6 ± 0.8 mm, *P* = .0404) or the 2 Gy-irradiated group (27.9 ± 0.6 mm, *P* = .0044). We attribute the greater terminal length of the both the nonirradiated control and 10 Gy-irradiated limbs to the elongation accumulated during the extended survival period in comparison to the nonirradiated and 2 Gy groups.

## 4. Discussion

### 4.1. Model Development and Refinement

Subcutaneous inoculation of the tumor cells is a classical approach used to study the *in vivo* response of the tumors to experimental therapies. The translational relevance of ectopic xenograft studies can be problematic, as the placement of tumor cells into a physiologically foreign environment may not faithfully recapitulate the physiologic microenvironment of the primary lesion, adjacent nonneoplastic stromal and parenchmyal cells, or local vasculature [12–16]. Similarly, intravenous and intracardiac injection models fail to account for the initial events that lead to metastasis and so fail to appreciate the importance of this aspect of tumor biology. Further, the response of an ectopic tumor to systemic or localized therapies may not realistically resemble that of a malignancy in its primary tissue of origin [17]. For these reasons, orthotopic placement is rapidly gaining favor among cancer researchers, but this approach is not without limitation. Orthotopic placement can require invasive procedures to achieve the engraftment and assessment of tumor growth in situ. This limitation can therefore require a large number of subjects to achieve statistical significance due to terminal data collection. Further, this approach can be relatively insensitive to occult lesions and systemic dissemination, which are essential to understanding this most critical aspect of cancer biology and treatment. 

Our primary objective in this series of experiments was to develop an orthotopic xenograft model that would permit parallel examination of tumor and epiphyseal responses to radiotherapy. Our initial attempts to establish an orthotopic tumor by injection of tumor cells in an aqueous suspension were not fruitful. In all likelihood, this reflected a failure of engraftment, which was addressed in subsequent refinement of our model. The enhancement of graft success by Matrigel suspension has been previously reported for a wide variety of tumor cell types [18, 19]. Our subsequent experiments involved suspension of rhabdomyosarcoma cells in a Matrigel basement matrix, which greatly enhanced tumor engraftment in comparison to aqueous inoculation media (Figure 1, Table 1). The precise mechanism by which the artificial matrix improves tumor cell survival and growth remains unclear but may involve sequestration from NK cells or induction of angiogenesis at the site of tumor implantation [20, 21]. There was no gross or microscopic evidence of residual Matrigel at 64 days following injection. Similarly, no residual matrix was observed in the tumor bed of mice inoculated with RD or RC13 cells at 84 days, suggesting that the matrix is resorbed in a manner that is independent of the presence of tumor cells. 

A second objective in developing this model was to measure tumor response to experimental therapies with the long term goal of testing complementary radioprotective agents with this model. Tumor growth can be assessed by measuring the dimensions of an externally evident tumor but are relatively insensitive and fail to account for occult lesions or systemic dissemination. The application of bioluminescence imaging technology, as demonstrated recently by Comstock et al. [22], and reviewed by Michelini et al. [23], greatly enhances the ability to measure orthotopic tumors in deep or inaccessible locations. Studies by Jenkins et al. [24] and Lim et al. [25] have demonstrated that luminescence imaging of tumor burden is much more sensitive to occult tumors than palpation or other external evidence. Previous work by Seitz et al. established a xenograft model involving alveolar rhabdomyosarcoma cells stably transfected to express the *DSred2* fluorescent protein [26] for localization of subcutaneously inoculated tumors. However, this earlier study quantified tumor burden using external physical dimensions, rather than the fluorescence signal intensity, presumably due to limits on the depth of penetrence of the excitation and emission wavelengths. Further, these luminescence-imaging systems allowed more precise quantitation of tumor burden than physical examination and were sensitive to decreases in tumor signal due to various physiological manifestations, including focal tumor necrosis. 

With these considerations in mind, we established two rhabdomyosarcoma cell lines which stably expressed firefly luciferase for use developing our xenograft model. We demonstrated that bioluminescence imaging is a useful modality for verifying tumor placement, engraftment, and subsequent evaluation of tumor growth and treatment response, as has been demonstrated for a wide variety of other solid tumors (reviewed in [25]). Through the use of this technology, we were able to serially quantify tumor growth and response to treatment. Further, we demonstrated that RC13-luc derived tumors are sensitive to a single high-dose fraction of X-radiation.

### 4.2. Comparison of Embryonal and Alveolar Rhabdomyosarcoma Xenografts

We observed little growth of RD-luc embryonal rhabdomyosarcoma tumors in either inoculated site (Figures 3(a)–3(c)), and there were no lesions suggestive of metastatic dissemination observed. Embryonal rhabdomyosarcoma variants tend to be somewhat less aggressive and are frequently associated with small muscles of the head, neck, orbit, and genitourinary tract of infants and young children, features that our model did not replicate. Orthotopic placement of a xenograft tumor was hypothesized to improve upon ectopic approaches as a more faithful replication of the physiologic conditions experienced in situ. Consistent with this hypothesis, we observed that orthotopic inoculation of RC13-luc alveolar rhabdomyosarcoma cells into the distal thigh musculature resulted in a much more aggressive tumor growth than when compared to subcutaneous inoculation (Figures 3(d)– 3(f)). Further, distant metastases were apparent in 3 of 16 nonirradiated mice bearing RC13-luc tumors suggesting that the inoculated cells were competent for systemic dissemination (Figures 4(a)–4(d)). Alveolar-variant rhabdomyosarcoma tends to be the more aggressive subtype, most frequently arising from the large muscles of the appendicular skeleton, adjacent to the growth plate of older children and adolescents. Each of these features was replicated in the mice bearing orthotopic tumors derived from the RC13-luc cell line. Differences in microenvironmental physiology including autocrine [27–29] or paracrine signals [30, 31], local hemodynamics, and tumor hypoxia [32, 33] at either subcutaneous or orthotopic inoculation site may be an important consideration but were not examined as contributing factors in this pilot study.

### 4.3. Radiation-Induced Physeal Bystander Effect in a Tumor-Bearing Animal Model

This model also allows examination of the clinical morbidity syndrome of limb length asymmetry and angular deformity that can occur when oncologic therapy mandates irradiation of an open physis. Using this model, we showed that the growth plate was sensitive to 2 Gy or 10 Gy radiation doses, producing significant limb length asymmetry. Of particular note was our finding that both limbs from the high-dose group were longer at termination than either of the sham-irradiated or 2 Gy-irradiated cohorts. We interpret this observation simply as a result of the prolonged survival associated with the higher radiotherapy dose. This supports previous observations reported in a rat model of unilateral radiation-induced limb length asymmetry [8, 34, 35]. suggesting that despite the initial growth plate injury, the growth plate retains some capacity for regeneration. These are important findings that support the clinical relevance of this small animal model for testing the safety and efficacy of radioprotectant and recovery-stimulating strategies. 

Certain limitations of this new model are evident but may be refined or modified to suit specific experimental applications in future work. For example, it is accepted those this model in its current inception deviates from clinical best practice standards of fractionated radiotherapy with 45–56 Gy administered as a series of 1.8–2 Gy exposures [36, 37]. Future refinements to address this limitation would be anticipated to resemble that observed previously in an analogous rat model developed by our laboratory, wherein a fractionated radiotherapy protocol would produce a more subtle reduction in limb length than an equivalent single exposure [38, 39]. Another potential limitation to this model is the question of whether xenograft models using immunocompromised host mice are immunologically relevant. This limitation is much more difficult to address, given the propensity for graft rejection in intact animals. Recent work [40, 41] demonstrating the spontaneous development of rhabdomyosarcoma tumors in mice with somatic PTCH1 haploinsufficiency suggests which may provide an ideal model to study rhabdomyosarcoma biology. However such a model may further complicate parallel studies of skeletal growth given the central role that PTCH1 plays in regulating growth plate chondrocyte biology. Despite these limitations, the current model appears to be a reasonable and clinically relevant means to study the role of novel complementary therapies, which will be explored in future work.

## 5. Conclusions

This series of experiments describes the development of a new orthotopic xenograft model of rhabdomyosarcoma for the study of tumor and bystander responses to radiotherapy. This work also presents variants of two well-established rhabdomyosarcoma cell lines which stably express firefly luciferase, allowing the use of *in vivo* bioluminescent imaging. Using this model system, we were able to demonstrate responses of tumor cells and bystander tissues that reasonably resemble clinical outcomes. We anticipate that this new model will be a valuable tool in evaluating the efficacy and safety of novel radioprotectant and radiorecovery strategies prior to their use in human clinical trials.

## Figures and Tables

**Table 1:** Experimental design: IM: intramuscular inoculation; SQ: subcutaneous inoculation; HBSS: Hank's buffered salt solution; Inoc.: approximate number of cells inoculated; RTX: radiation therapy; Gy: Gray, units of absorbed radiation; Term.: day of study when mice were euthanized; EOS: end of study; TB: euthanized due to tumor burden.

Cell line	N	Location	Media	Inoc.	RTX	Engraftment	Term.	Reason
RD	8	IM	HBSS	1 × 10^6^	—	0/6	84 d	EOS
RD	8	IM	HBSS	1 × 10^5^	—	0/6	84 d	EOS
RD	8	IM	HBSS	1 × 10^4^	—	0/6	84 d	EOS

RD-Luc	4	SQ	Matrigel	1 × 10^6^	—	4/4	64 d	EOS
RD-Luc	4	IM	Matrigel	1 × 10^6^	—	4/4	64 d	EOS
—	2	SQ	Matrigel	—	—	0/2	64 d	EOS
—	2	IM	Matrigel	—	—	0/2	64 d	EOS

RC13-Luc	6	SQ	Matrigel	1 × 10^6^	—	5/6	43 d	TB/EOS
RC13-Luc	6	IM	Matrigel	1 × 10^6^	—	6/6	35–43 d	TB/EOS

RC13-Luc	4	IM	Matrigel	1 × 10^6^	0 Gy	4/4	56–64 d	TB
RC13-Luc	6	IM	Matrigel	1 × 10^6^	2 Gy	6/6	57–64 d	TB
RC13-Luc	6	IM	Matrigel	1 × 10^6^	10 Gy	6/6	57–84 d	TB/EOS

**Table 2:** Terminal femur, tibial, and total limb length. Data shown are mean ± 1SD; ^‡^differences between control and irradiated, tumor-bearing limbs were accepted as significant when *P* ≤ .05 by Student's paired *t*-test.

	Femur length (mm)		Tibia length (mm)		Limb length (mm)	
	Control	Irradiated	Control	Irradiated	Control	Irradiated
0 Gy						
Mean ± SD	13.5 ± 0.6	13.2 ± 0.5	15.1 ± 0.6	15.1 ± 0.8	28.6 ± 0.8	28.3 ± 0.9
*P^‡^*	*P* = .0892		*P* = .6467		*P* = .3769	
2 Gy						
Mean ± SD	13.4 ± 0.4	12.8 ± 0.6	15.6 ± 0.4	14.9 ± 0.4	29.1 ± 0.7	27.9 ± 0.6
*P^‡^*	*P* = .0141		*P* = .0156		*P* = .0072	
10 Gy						
Mean ± SD	14.3 ± 0.4	13.8 ± 0.6	16.1 ± 0.3	15.6 ± 0.3	30.4 ± 0.6	29.4 ± 0.8
*P^‡^*	*P* = .0013		*P* = .0123		*P* = .0003	
